# Supplementary material for: Are carbon emissions trading and green financial instruments synergistic? -Comprehensive quantitative research based on content analysis
Source: PLoS One. 2024 Mar 7;19(3):e0298601. doi: 10.1371/journal.pone.0298601 (PMC10919601; doi:10.1371/journal.pone.0298601)
Supplement: S1 Data — (ZIP) [file pone.0298601.s001.zip › Data/地级市Z_F工作报告.zip!/╒π╜¡/╜≡╗¬╩╨/└┤╫╘╣1⁄2╓┌║┼í╛┬φ┐╦╩2╛▌═°í┐_2016.docx]

各位代表：

现在，我代表市人民政府向大会作政府工作报告，请予审议，并请市政协委员和其他列席人员提出意见。

一、2015年主要工作和“十二五”发展成就

　　2015年是不平凡的一年。面对严峻的宏观环境和繁重的发展任务，市政府按照“三严三实”要求，始终保持奋发有为的精神状态，认真贯彻落实市委各项决策部署，较好地完成了市六届人大六次会议确定的各项目标任务。

（一）注重提质增效，保持经济平稳发展

　　加快发展现代生态循环农业。新建省级粮食生产功能区12万亩、省级现代农业综合园区5个、设施农业1.1万亩，新增省级示范性家庭农场24家、市级规范性农民专业合作社32家、市级以上农业龙头企业20家。

加快推进工业经济转型升级。“五大千亿”产业实现规上产值（收入）4279亿元，占规上工业产值的86.8%。完成工业技改投资550亿元、增长24.5%，实施“机器换人”重点项目230项，新增“个转企”2030家、“小升规”411家、“规改股”75家、上市公司2家、“新三板”挂牌企业13家、中国驰名商标10件。兰溪老工业基地振兴取得突破。华科汽车、吉利发动机实现当年建设、当年投产。众泰集团汽车销售超百亿元。新能源汽车推广应用居全省第二。加强企业“两链”风险防范和处置工作。建筑工业化加快推进，建筑业增加值增长9.9%。

加快现代服务业集聚发展。重视抓好会展业，举办了一系列国际性、全国性、地区性展会。引进中兴通讯、科大讯飞、奇虎360等企业，北大信息科技园等开园运营。全市实现电子商务交易额3360亿元，网络零售额1344亿元，分别增长42.6%和58.4%。旅游业加快发展，实现旅游收入756亿元，增长22%。横店影视城接待游客突破1500万人次。义乌中国小商品城、永康中国科技五金城等5家市场获评全国诚信示范市场。

2015年，全市实现生产总值3406亿元，增长7.8%；一般公共预算收入309亿元，增长8.8%；固定资产投资1836亿元，增长15.1%；社会消费品零售总额1783亿元，增长12%；城乡居民人均可支配收入分别达到43193元和20297元，增长8.5%和9.5%。

（二）深化改革开放，增添发展活力

　　荣获2015中国“一带一路”最具活力城市称号。“义新欧”班列实现双向常态化运行，金义综合保税区获国务院批准建设，进口肉类指定口岸获批筹建，金华至宁波海铁联运暨金华至中亚、中俄国际班列和义乌至伊朗国际班列、兰溪嘉宝物流班列开通。实际利用外资2.7亿美元，引进浙（婺）商回归产业项目资金261亿元，实现外贸出口476亿美元，增长20.2%，其中出口“一带一路”沿线国家增长24.7%。

义乌国际贸易综合改革深入实施，国内贸易综合改革试点正式启动，国际邮件互换局投入使用。全国现代服务业综合试点绩效评价居全国第二。低丘缓坡综合开发利用试点、国家跨境电商服务试点取得新成效。浦江扎实开展全省协调推进“四个全面”改革试点。义乌列入国家新型城镇化试点，东阳横店开展国家中小城市综合改革试点。

加快实施经济体制改革，新增各类市场主体11.7万户。商事制度改革提速，实现“三证合一”、“五证合一”到“一照一码”。扎实推进“四破攻坚”专项整治。东阳、义乌、浦江省级资源要素市场化配置改革试点顺利实施。开展不动产统一登记。加快农村“三权”改革，浦江深化集体林权制度改革经验在全国推广。

（三）打好“组合拳”，提升发展质量

　　加力“五水共治”，全面消除金华江流域劣Ⅴ类水质断面。出台实施污水处理“金华标准”，加快截污纳管等十大工程建设。整治市控排污口70个，提升整治黑臭河625公里，新建城镇污水管网732公里，新增污水处理能力21万吨，提标改造一级A城镇污水处理厂16座，完成治水总投资125亿元。10个市界出境断面全部实现Ⅲ类水质以上，20个县（市）交接断面Ⅲ类水质以上达17个，42个地表水断面Ⅲ类水质以上28个。划定生态红线区5618平方公里。完成病险水库除险加固25座，市第二水厂主体工程土建完工，农村饮水安全工程受惠人口14.5万人。

大力推进“三改一拆”、“四边三化”、“气尘合治”和治堵治超。完成“两路两侧”整治任务，实施拆违1536万平方米、“三改”2129万平方米，全国违法建设治理现场会在我市召开。启动城中村改造80个，完善撤村建居25个，改善市政基础设施10个，建成城中村改造公寓式安置房409万平方米。淘汰黄标车4万多辆、燃煤锅炉2126台，金华市区PM2.5浓度下降15.7%，空气优良天数264天、比上年增加29天。打通断头路10条，金华市区一环路、二环路全线贯通。金华市区公交分担率提高2.5个百分点。全市新增停车位2.4万个、投放公共自行车3.1万辆，查处违法超限超载运输车辆7975辆。

坚持治转结合，提升发展质量。新增国家级高新技术企业68家、省级企业研究院11家、授权发明专利1015项，高新技术产业增加值比重达31%，新增院士专家工作站5家、入选国家“****”4人，打造“青年创业谷”。义乌丝路金融小镇、武义温泉小镇、磐安江南药镇和金华开发区新能源汽车小镇、东阳木雕小镇、永康赫灵方岩小镇入围省级特色小镇创建名单。获批创建全国质量强市示范城市。

（四）加强城乡统筹，增强发展合力

　　编制完成金华—义乌都市区规划纲要，优化“一轴两带多组团”空间布局。加快中心城区建设，多湖中央商务区完成4个村（社区）房屋征收工作，道路框架、综合管廊建设、地下空间开发等加快推进，总部中心等10个项目开工建设。婺城二七区块棚户区改造房屋征迁基本完成。实施“三江六岸”景观提升工程，燕尾洲公园被评为2015年度世界建筑节最佳景观奖。金义都市新区加快核心区块建设，引进油电混合动力变速箱、传化物流信息港等大项目。义乌丝路新区、陆港新区、科创新区加快建设，国际电子商务城一期、快递企业集聚中心、普洛斯物流园等项目顺利实施。

加快美丽乡村建设。建成美丽乡村风景线21条，创建精品村43个、秀美村88个，新增省级生态乡镇18个、美丽宜居示范村12个。义乌荣获国家森林城市称号，东阳花园获评中国美丽乡村建设示范村，兰溪芝堰开展国家级美丽宜居示范村创建。加速农村“三治”，75%的村庄达到洁美村标准，完成赤膊房整治822万平方米，农村生活污水治理和生活垃圾分类减量处理行政村覆盖率分别达80%和72%。“两次四分”农村生活垃圾分类减量处理办法全省推广，武义获评全国农村生活污水全面治理示范县。加强历史文化村落保护开发，浦江嵩溪荣获第六届“中国景观村落”称号。

加快推进城乡基础设施建设。金温快速铁路、东永高速公路建成通车，金台铁路、义乌疏港高速、杭绍金台高速、杭金衢高速拓宽改造和一批国省道干线公路改扩建项目加快实施。衢江航运开发进展顺利，浙中公铁水联运港项目规划建设有序推进。金兰、金武城际公交相继开通。

（五）强化民生保障，提高群众获得感

　　实施精准扶贫，全面消除年人均收入4600元以下贫困现象。健全职工工资稳定增长机制，最低工资标准提高12.9%，企业退休人员基本养老金标准提高13%，城乡居民基础养老金最低标准提高20%，被征地农民基础保险金标准提高12.1%，城乡最低生活保障标准分别提高11.8%和20.9%。

提升公共服务水平。8890平台累计受理市民诉求64.3万件，开设了虚拟养老院和光明电影院。统筹推进各类教育改革发展，义务教育标准化学校创建率达86.9%，重视解决进城务工人员子女“就学难”问题，加强住宅小区配套幼儿园建设，市特殊教育中心建成使用。深化公立医院改革，全面推行分级诊疗制度，推进“双下沉、两提升”，市中心医院东扩工程完工。市博物馆对外开放，新增农村文化礼堂203家。体育健身运动广泛开展，290所学校体育场地设施向社会开放。新增机构养老床位2252张、家院互融养老服务机构22家、城乡居家养老服务照料中心847家，金东农村居家养老模式入选“2015中国民生示范工程”。城市文明程度指数测评居全省第四。

创新社会治理。加强社会治安防控体系建设，重大刑事案件破案率达97.7%，荣获省平安建设“银鼎”。健全食品药品安全监管机制，新建农产品安全快速检测室82个、放心农贸市场32家。加强安全生产监管，安全生产事故起数、死亡人数同比分别下降6.5%和2.1%。开展信访百日攻坚活动，重视抓好初信初访和矛盾一线化解，创新人民调解工作，加强社区矫正和公共法律服务等工作。

各位代表，“十二五”时期是我市坚持“发展城市群、共建大金华”战略主线、奋力追赶的五年，是围绕“百姓富裕、浙中崛起”目标、全面建设小康社会的五年。

——五年来，综合实力迈上新台阶。生产总值占全省比重从7.6%提高到7.9%，人均生产总值达71478元，一般公共预算收入年均增长14.7%，城乡居民人均可支配收入年均分别增长10.7%和12.1%，城乡收入差距缩小。“十二五”期间，在年度监测的28项主要经济指标中，25项指标在全省占比提高。

——五年来，转型升级取得新进展。三次产业实现“二三一”向“三二一”转变，服务业占比达50.7%，提高7.6个百分点。建成省级粮食生产功能区61.7万亩、省级现代农业综合园区11个。工业发展实现晋位，列全省第六，培育超百亿产业集群18个、规上产值超20亿元工业强镇51个。新增国家级高新技术企业229家、上市公司10家。累计完成固定资产投资6785亿元、年均增长18.9%，社会消费品零售总额年均增长14.2%。

——五年来，改革开放取得新成效。累计实现外贸出口1563亿美元，年均增长31.4%，从全省第七跃居第三，占全省比重达到17.2%，5年提高10.4个百分点。累计实现进口76.6亿美元，引进外资12.4亿美元。国际贸易、现代服务业、低丘缓坡开发、影视文化实验区等国家及省部级改革试点成效明显。

——五年来，环境治理取得新突破。在全省率先打响“五水共治”大会战，近三年治水投入295亿元，消灭垃圾河1090公里、黑臭河2003公里，荣获2014年全省治水“大禹鼎”。实施拆违4847万平方米、“三改”4544万平方米。空气质量优良天数逐年增多，群众满意度连续两年上升。金华市区获评国家园林城市，义乌、磐安获评国家生态县（市），金东、东阳、义乌、永康、磐安获评省“美丽乡村创建先进县”，浦江入选全省首批“最具魅力新水乡”。

——五年来，城乡发展呈现新面貌。坚持金华市区和义乌“双核”融合发展，设立金义都市新区、多湖中央商务区、金华山旅游经济区，义乌城乡新社区建设纵深推进，各县（市）中心城区、小城市、中心镇、特色镇、美丽乡村加快建设，城市化率达64.5%，提高5.5个百分点。基础设施互联互通、共建共享加快推进，金华跨入“高铁时代”，县（市）间基本形成“一小时交通圈”。

——五年来，民生改善迈出新步伐。义务教育入学率和巩固率达100%，高中段教育毛入学率98.6%，高等教育毛入学率56.3%。城镇登记失业率控制在2.9%以下，养老、医疗参保率分别达95.5%和98%。建成居家养老照料中心2837家，惠及20万老年人。保障性安居房竣工3.8万套。建成农村文化礼堂644个。出生人口性别比实现五连降。安全生产事故起数、死亡人数持续下降，连续五年入选“中国30个最安全城市”。

——五年来，政府自身建设得到新加强。认真开展党的群众路线教育实践活动和“三严三实”专题教育，深入开展“五员五送”、“百企万人评机关”等活动。完善“四张清单一张网”，开通“微政务”，实施公共资源招投标领域“红、黑”名单制度，“零地”建设项目从立项到开工压减至50个工作日。建立健全市政府常务会议学法、政府法律顾问、人大代表政协委员列席市政府常务会议等制度，行政诉讼败诉率降至6.4%。义乌首设行政复议局。认真落实人大及其常委会决议决定，自觉接受人大法律监督和工作监督、政协民主监督、群众社会监督。加强审计监督，促进增收节支20多亿元。“三公”经费支出年均下降23%。

全面发展老龄、残疾人和关心下一代等事业，广泛开展“爱心红手环”等慈善活动。支持工会、共青团、妇联等人民团体开展工作，加强民族、宗教、外侨、对台、统计、气象、民防、档案、地方志、仲裁、应急管理等工作。支持国防和军队建设，实现全国双拥模范城“三连冠”。深化援疆、援藏、援青及“双对口”工作。推进“六五”普法。完成第三次经济普查和第六次人口普查。

各位代表，“十二五”发展成绩来之不易。这是省委、省政府和中共金华市委正确领导的结果，是全市人民创业创新、奋力拼搏的结果。在此，我代表市人民政府，向全市人民和外来投资者、建设者表示崇高的敬意和衷心的感谢！向人大代表、政协委员，向离退休老干部，向各民主党派、工商联、人民团体、无党派人士，向驻金部队、武警官兵、部省属驻金单位，表示衷心的感谢！向所有关心、支持我市建设和发展的港澳台同胞、海外侨胞以及社会各界朋友，表示衷心的感谢！

我们也清醒地看到，我市经济社会发展和政府工作还存在一些问题。主要有：经济下行压力较大，部分企业转型升级仍然困难，金融风险潜在隐患仍然较多；产业层次不够高，创新能力不够强，科研机构、创新企业、创新人才不够多；中心城区辐射带动能力不够强，都市区统筹发展有待进一步加强；资源约束趋紧，环境承载力下降，治水治气治堵任务依然艰巨；基本公共服务供给、保障和改善民生的任务还比较重，人口老龄化压力增大；生产安全、食品安全、网络安全还存在薄弱环节，维护社会稳定面临新的挑战；政府职能转变还不到位，依法办事素养和能力有待加强，重审批轻监管、重执法轻服务现象仍然存在。市民卡工程、薄弱学校改造、建筑渣土资源化利用处置场建设等3个民生实事项目未全面完成。对此，我们一定高度重视，采取更加有力措施认真加以解决。

二、“十三五”发展主要目标任务

　　“十三五”是金华实现追赶发展、高水平全面建成小康社会的关键时期。根据中共金华市委《关于制定金华市国民经济和社会发展第十三个五年规划的建议》，市政府制定了《金华市国民经济和社会发展第十三个五年规划纲要（草案）》，提请大会审议。经本次大会批准后，市政府将认真组织实施。

“十三五”发展的指导思想是，以邓小平理论、“三个代表”重要思想、科学发展观为指导，深入贯彻习近平总书记系列重要讲话精神，以“四个全面”战略布局为统领，以“八八战略”为总纲，以“把握新常态、共建都市区”为战略主线，按照“走在前列、共建金华”的要求，坚持发展第一要务，坚持“五大发展”理念，坚持高质高效发展不动摇，着力加强结构性改革，加快形成引领经济发展新常态的体制机制和发展方式，加快形成县域经济向都市区经济转型的发展格局，加快形成全省发展新的重要增长极，全力打造“丝路枢纽、商贸之都、智造强市、文化名城、旅居福地”，高水平全面建成小康社会，努力开创“两富”“两美”金华建设新局面。

　　“十三五”发展的主要目标是，全市生产总值年均增长7.5%—8%，城乡居民收入增长与经济增长同步。到2020年，人均生产总值10万元左右，居民人均可支配收入超5万元，社会保障覆盖率达100%，研发经费支出占生产总值比重达2.5%左右，高等教育毛入学率达62%，综合实力明显增强，生活水平明显提升，城乡统筹明显加强，生态环境明显改善，治理体系明显健全，努力建成走在全省前列的城市。

加快“十三五”发展、实现“十三五”目标，必须坚持“五大发展”理念，发挥优势、补齐短板，集中精力抓好既该干又能干成的大事。

　（一）着力突出制度供给，在激发市场活力上走在前列

　　加强政府改革创新。深化“四张清单一张网”改革，大力推进简政放权，实现行政管理和公共服务“一站式”在线运行，着力破解“事难办”问题。理顺政府与市场、社会的关系，市场的交给市场，企业的交还企业，社会的委托社会。加快打造“互联网+”政务先行区，建设“政务云”，提高“微政务”应用水平。完善全口径政府债务管理，扩大政府购买公共服务范围。

放大改革试点效应。统筹推进义乌国际贸易、国内贸易、新型城镇化和农村改革试验区等国家改革试点，深入推进国家现代服务业综合试点，形成可复制、可推广的经验和做法。扎实开展浦江协调推进“四个全面”战略布局省级试点，努力建设浦江国际水晶产销中心。加快推进横店国家中小城市综合改革试点，全力打造国际影视名城、特色旅游休闲小镇。加快建设质量强市示范城市。

创新要素配置机制。拓宽重点投资建设项目行政审批绿色通道，进一步清理规范中介服务和涉企收费，建立“区域环评、能评+区块能耗、环境标准”新机制，逐步实行企业独立选址项目高效审批、非独立选址项目不再审批。推进土地、能源、环境容量、水资源等要素市场化配置改革，全面建立阶梯价格制度。到2020年，节能节地节水、环境、技术、质量、安全等市场准入标准覆盖所有新建项目和存量企业。创新城市基础设施建设和政府社会资本合作（PPP）机制。

（二）着力实施创新驱动，在转换发展动力上走在前列

　　强化科技创新。加快构建以企业为主体的科技创新体系，以政商产学研金为一体的区域创新体系，以高层次人才、高技能人才为重点的“蓝海”支撑体系，以大学生创业为主体的众创发展体系和以产业引导基金为主导的金融服务体系。力争市级以上高新技术企业达1200家，其中国家级750家，省级科技型中小企业2000家，“****”和“双龙计划”人才各200人以上，加快打造“青年创业谷”。

强化产业创新。以构建现代产业体系为目标，加快实施“五大千亿”产业培育行动，企业扶优扶强行动，“四换三名”、“两化”融合、智能制造行动，中小企业发展及高成长企业培育行动和对接多层次资本市场行动。到2020年“五大千亿”产业总产值（收入）突破2万亿元，在役工业机器人超1万台，培育销售收入超百亿元企业8家、“浙江制造”标准50个以上。大力引导企业开展股份制改造和兼并重组，通过多层次资本市场直接融资。

强化平台创新。加快中科院金华科技园、北大信息科技园、金华（国际）科技城等平台建设，支持县市建设全面创新改革试验区。实施省级产业集聚区、省级高新技术园区和省级以上开发区整合提升，加快建设省级现代服务业平台。大力推进特色小镇、工业强镇和电商新镇建设，到2020年，建成省级特色小镇10个，市级特色小镇30个左右，累计投资超1000亿元，争创省十强工业强镇2个，培育电商新镇50个。

　（三）着力实现优势互补，在共建都市区上走在前列

　　强化共建共融共享共赢理念。按照建设全省第四大都市区的目标，加快编制金华—义乌都市区规划，推进“多规合一”，加快形成“一轴两带多组团”都市区空间发展新格局，把八婺金华打造成利益共同体。

加强都市区联动发展。加快建设中心城区，提升中心城市能级。多湖中央商务区要加快建设“五大中心”，金义都市新区要基本建成“田园智城、电商新城、都市新区”。加快推进湖海塘、尖峰山、铁路客站、婺州古城、三江六岸等重点区块和金华山旅游经济区、婺城新城区、金东新城区开发建设。义乌加快丝路新区、陆港新区、科创新区建设。完善县域中心城市节点功能，提高创造就业岗位、承载农民转移能力。强化中心镇及小城镇基础作用，积极承接城市基础设施和公共服务，并向农村延伸，提高服务“三农”能力。

加强都市区聚合发展。优化现代产业空间布局，以金义主轴和金兰永武产业带为重点发展先进装备制造产业，以金华市区和兰溪、东阳、武义为重点发展健康生物医药产业，全域发展信息经济、休闲旅游服务和文化影视时尚产业。加快打造综合交通枢纽，全力构建“两高（高速公路、高速铁路）三快（城际快速干线、快速公交、快速轻轨）五大（大枢纽、大路网、大物流、大口岸、大走廊）”为骨干的现代综合交通体系，完成综合交通投资1500亿元以上。

加快城乡一体化发展。推进“一区一镇”建设，打造农业“两区”升级版。提升农业科技创新和设施装备水平，构建现代农业产业体系，全市粮食播种面积稳定在183万亩。加快推进小城市、特色小镇、中心城镇、卫星城镇发展，强化与城区的产业协作协同，提高城镇产业发展和集聚人口能力，推进农民就地市民化。加强农村交通、环保、通信等基础设施建设，加快农村“三权”改革和户籍制度改革。

推进军民融合深度发展。完善军民融合发展工作机制，建设一批军民融合产业基地，培育一批重点企业，打造共享平台。全力支持驻金部队和武警部队建设，做好国防动员、后备力量建设、国防教育、人民防空和双拥优抚安置等工作。

（四）着力打造绿色生态，在建设美丽浙江上走在前列

　　实施美丽金华建设行动。紧扣8大绿色发展目标，深入开展“五水共治”、“三改一拆”等11项行动，到2020年基本建成生态市和全省生态文明示范区、美丽浙江先行区。强化制度保障，整合生态环保财政政策，完善主要污染物排放财政收费制度，落实最严格的水资源利用管理、耕地保护和能源、环境容量“双控”制度，实行污水排放、工业废气排放总量控制。划定森林、湿地、物种生态保护红线，健全“绿水青山就是金山银山”的体制机制。

加大生态环境治理力度。统筹推进治水治气、治城治乡、治土治山，切实加强环境保护、生态修复。全面实施污水处理“金华标准”和城乡生活垃圾分类减量处理。到2020年城镇污水处理厂全部达到一级A排放标准，县（市）交接断面和省控断面水质Ⅲ类以上比例达到90%和75%。加快棚户区、城中村、景中村和城乡危旧房改造，实现所有县（市、区）基本无违建。持续开展“气尘合治”，PM2.5平均浓度下降40%，环境空气质量优良天数比例达85%。健全治堵治超长效机制，金华市区公交分担率超25%。有效控制农业“两区”土壤污染问题。积极推进建筑垃圾、餐厨垃圾资源化利用，提升危化废弃物无害化处理水平。

发展绿色低碳经济。加大高耗能、重污染企业整治力度，推行清洁生产，积极培育循环经济和节能环保产业，省级以上工业园区全部建成生态工业园区。开展生态循环农业示范县创建，推广种养、农牧、林牧结合的生态循环农业模式，扶持发展创意农业、休闲农业、景观农业、养生农业。积极发展新能源汽车，到2020年全市公交车、出租车、营运专车、一般公务用车替换为新能源汽车或改造为清洁能源汽车。大力发展绿色建筑产业，拉长产业链，建成绿色建筑示范区100个以上。

建设美丽城镇、美丽乡村。加强城乡基础设施和服务配套建设，促进有机更新、环境改善、景观再造。巩固国家卫生城市、国家园林城市创建成果，争创全国文明城市。稳定提高森林覆盖率，创建森林城市群。深入推进“千村示范、万村整治”工程，实施美丽婺州民居计划，重视历史文化村落保护利用，创建旅游风情小镇10个、美丽乡村精品村150个、民宿特色村20个。

（五）着力推进先行先试，在提升开放层次上走在前列

　　构建开放大通道。充分发挥“义新欧”班列、义乌国际邮件互换局作用，加快建设“义甬舟”开放大通道，推进义乌小商品线上、线下“两个万亿级”市场建设。大力开展“金华万企进电商”和“农村电商三年行动计划”，加快拓展网上门户开放通道。依托在金高校和科研机构，打造“一带一路”国际研学交流基地，深化“海外名校学子走进金华古村落”活动，拓宽民间开放交流通道。积极参与长江经济带和浙皖闽赣国家东部生态旅游试验区建设，推进山海协作，重视援疆、援藏、援青及“双对口”工作，扩大国内区域合作发展通道。

打造开放大平台。充分发挥义乌保税物流中心（B型）作用，全力做好金义综保区建设、招商、投融资、运营等各项工作，努力打造国内一流的跨境综合服务区、最佳跨境电商生态圈、“买全球卖全球”线上线下融合大平台。加快浙中公铁水联运港、义乌国际陆港、东阳国际物流港、兰溪嘉宝物流港、义乌机场空港、永康无水港等物流产业集聚区建设，强化陆港口岸功能，进一步推进海关、检验检疫通关一体化。

形成开放大格局。厚植出口优势，拉长进口短板，进一步提高进出口占全省的份额。围绕“五大千亿”产业，实施招商引资“五年倍增计划”。坚持“引进来”与“走出去”并重，引导民营企业沿“一带一路”集团式、产业链式走出去，建立生产基地，开设“海外仓”，承接土建路桥等海外工程，设立产业合作园区，开辟国际市场。

（六）着力谋求人民幸福，在创造美好生活上走在前列

　　促进城乡居民普遍持续增收。加大精准扶贫、脱贫力度，切实解决脱贫群体“返贫”问题，大力推进电商扶贫、旅游扶贫、光伏扶贫、异地搬迁等帮扶造血工程。落实就业优先战略，完善收入分配制度和劳动报酬增长机制，逐步提高最低工资标准，新增城镇就业35万人，确保2020年城乡居民人均可支配收入比2010年翻一番，农村居民人均可支配收入突破3万元，城乡收入差距进一步缩小。

推动城乡公共服务均等化。高标准普及15年教育，支持高等院校、科研机构推进产教融合、校企合作，建设众创空间和平台。深化医疗卫生体制改革，提高“双下沉、两提升”实效，完善药品采购定价和监管机制，每千人口执业（助理）医师数达3.2人。加快推进养老保险制度互转和医疗保险制度统筹整合，实现社保人群全覆盖。重视社会养老服务，千名老年人口养老床位数达50张。健全基本公共文化服务体系，坚持“建、管、用”结合，加强农村文化礼堂建设，创新设立网络图书馆、网络博物馆、网络剧场等网上文化场所，加强文化遗产保护利用。大力推进全民健身。统筹做好妇女儿童、老龄、残疾人等工作。

加强和创新社会治理。深化“枫桥经验”、“后陈经验”，健全群众诉求表达、利益协调、矛盾调处和权益保障机制，加强立体化社会治安防控体系建设，提升生产安全、食品安全、网络安全治理能力，确保社会和谐稳定。深入实施公共资源招投标领域“红、黑”名单制度，设立企业信用联合奖惩平台，完善以企业为主体的社会诚信体系，建设信义金华。发挥“最美现象”、家规家训家风等传播正能量作用，探索建立城乡社区、社会组织、社会工作联动机制，形成法治、德治、自治三位一体治理新格局。

各位代表，高水平全面建成小康社会的目标任务十分繁重。我们坚信，在中共金华市委的坚强领导下，经过全市人民的共同努力，“十三五”发展的宏伟蓝图一定能够实现！

三、2016年主要工作

　　2016年是“十三五”开局之年，做好今年的工作对“十三五”发展至关重要。当前，国际形势错综复杂，不确定因素较多。国内经济形势总体向好，但下行压力依然较大。综合分析各方面因素，建议今年全市经济社会发展的主要预期目标为：生产总值增长7.5%—8%，一般公共预算收入增长8%左右，城乡居民人均可支配收入增长与经济增长同步，去产能、去库存、去杠杆、降成本、补短板取得有效进展，节能减排降碳等指标完成省定目标任务。

做好2016年工作，必须以党的十八大和十八届三中、四中、五中全会精神和习近平总书记系列重要讲话精神为指针，全面贯彻落实中央、省委省政府和市委决策部署，牢固树立“五大发展”理念，适应经济发展新常态，坚持稳中求进总基调，以提高经济发展质量和效益为中心，着力加强结构性改革、强化创新驱动、打好转型升级组合拳，战略上打持久战，战术上打歼灭战，突出抓好转方式、促改革、抓统筹、强整治、惠民生、防风险等工作，为实现“走在前列、共建金华”开好局、起好步。

　　在具体工作中，坚持把加强供给侧结构性改革放在突出位置，着力抓好以下7方面重点：

（一）深化改革开放。统筹推进各项改革，扩大对外开放，增强经济发展的活力和动力。

加快“三去一降一补”。坚持工业强市战略不动摇，深化“四破攻坚”专项行动，建立健全优胜劣汰机制，积极稳妥处置“僵尸企业”，多兼并重组、多债务重组、少破产清算，实现市场出清。善待企业、关心企业、帮扶企业，打好“组合拳”，帮助实体经济降低企业成本。开展金融风险专项整治，加大逃废债打击力度，积极防范化解企业“两链”风险。坚持化解库存和稳定房价并举，合理有序安排土地出让，加大棚户区、城中村改造力度，强化基础设施配套，促进房地产业健康可持续发展。

深化各项改革。以习近平总书记批示要求“学习推广义乌发展经验”十周年为契机，深化义乌国际国内贸易、浦江协调推进“四个全面”战略布局和现代服务业综合改革等40多项国家、省部级改革试点。完善资源环境产权制度改革，加快财税体制改革，深化国资国企改革，整合提升公共资源交易平台，完善农村产权交易制度。

扩大对外开放。主动对接“一带一路”国家战略，加快构建“义新欧”、“义甬舟”开放大通道。全力抓好金义综保区建设，如期通过国家验收，并封关运行。发挥义乌国际邮件互换局作用，加强与国内知名电商平台合作，加快省级跨境电子商务园建设。强化招商选资，实际利用外资3.3亿美元、内资400亿元、浙（婺）商回归资金285亿元。

（二）推进科技创新。实施创新驱动首位战略，充分发挥科技创新在全面创新中的引领作用。

加大科技投入。支持企业加大研发投入，加大人力资本投资力度，发挥产业基金撬动作用。企业研发经费支出超100亿元，教育培训等人力资本投资超100亿元，科技型中小企业获得创业风险等投资超50亿元。

集聚创新人才。完善高层次人才、高技能人才政策服务体系，加大领军型团队引进培育力度，实施“院士智汇集聚工程”，力争培养引进“****”10人以上、“双龙计划”人才项目25个以上，新建院士专家工作站3个。

培育创新主体。新增国家级高新技术企业70家以上、企业研究院等研发机构100家以上、授权发明专利730件以上。发挥浙师大、上海财大浙江学院、金职院、义乌工商学院等在金高校人才资源优势，新增创新企业24家、省级科技型中小企业180家。

建设创新平台。整合提升省级高新技术园区和产业集聚区，加快中科院金华科技园、北大信息科技园、金华高新技术产业园、金华新能源汽车产业园、金华健康生物医药产业园及义乌中欧智造园、武义科技城、永康现代农机装备产业园、兰溪科技孵化园等科技平台建设。加快推进特色小镇建设，完成投资300亿元以上。

创新体制机制。深化与科大讯飞、中兴通讯、赛伯乐、保利集团、张江高科等知名企业战略合作，加快金华科技大市场建设，转移转化高校院所科技成果100项以上，推广使用创新券1000万元以上。

（三）加快产业优化升级。把发展实体经济放在更加突出的位置，努力保持经济平稳较快发展。

大力发展“五大千亿”产业。信息经济产业总产值增长22%，先进装备制造产业增长10%，健康生物医药和文化影视时尚产业均增长12%，休闲旅游服务产业增长15%。同时，传承发展火腿、木雕等历史经典产业。推进新型建筑工业化，促进建筑业转型发展、做大做强。

积极培育壮大市场主体。大力推进“四换三名”、“两化”融合、智能制造，加大龙头骨干企业培育力度，持续推进“个转企、小升规、规改股、股上市”。重点培育大企业大集团、“三名”试点企业、“两化”融合示范企业和高成长企业各100家以上，新增“小升规”企业300家、股份公司100家以上、上市和挂牌50家以上，新增工业机器人1000台，制定实施“浙江制造”标准10个。

继续扩大有效需求。优化投资结构，加大有效投资力度，固定资产投资增长15%以上，其中重大基础设施项目、重大产业项目、高新技术产业、生态环保投资均增长20%。坚持做强出口、补齐进口短板，全市外贸进出口增长10%左右。积极培育新的消费热点，大力发展电子商务，扩大信息、旅游、文化、健康等服务消费和新能源汽车消费，社会消费品零售总额增长10%左右。

（四）加强城市工作。坚持全市“一盘棋”，建立健全都市区统筹协调机制，加快都市区融合发展。

突出城市规划设计和建设管理。启动新一轮城市总规前期研究工作，开展土地利用总规修编，加快城市核心区、重点区块及重点项目城市设计全覆盖。加强城市管理，巩固国家卫生城市、园林城市创建成果，深化精品城市建设。加强城市有机更新，实施城中村改造30个，完善撤村建居13个，改造背街小巷19条。加快智慧城市建设。编制绿色建筑专项规划，推进绿色建筑产业发展。推进无障碍设施建设。加强城市地下空间开发，建设综合管廊，推进“海绵城市”建设。

突出城市功能区建设。多湖中央商务区开工建设道路8条、市科技文化中心（城市规划馆）等项目19个。加快金义都市新区产城联动步伐，完善基础设施和服务配套，启动项目建设125个。加快实施金华山开发三年行动计划，加强景区综合治理，推进景村一体化建设。加快二七区块、石门农场、湖海塘、婺州古城、高铁经济新组团、国际物流园区等功能区块规划建设。义乌加快打造现代化国际商贸城市。兰溪加快老工业基地振兴步伐，东阳推进文化产业全域化发展，永康建设浙江先进制造业基地，浦江建设世界水晶之都，武义建设温泉养生名城，磐安建设江南生态养生源。

突出基础设施互联互通、共建共享。加快建设金台铁路、金甬铁路、杭绍金台高速、义乌疏港高速和衢江航运开发工程，开工建设临金高速和351国道（浦江段）、39省道（东阳段）等一批国省道干线公路改建项目，以及金兰中线、金武快速路、金义永等城际快速通道，推进杭温高铁、金义东城际轨道、浦义东永高速（义乌—东阳段）等项目前期。建成横店通用机场，加快义乌机场提升扩能。完善县（市）间快速公交网络，开通运行BRT3号、4号线。建成市第二水厂，启动建设市第三水厂。

（五）加强“三农”工作。全面提升农业农村发展水平，促进农民持续增收。

积极推进农业现代化。加快农业机械化、信息化、标准化步伐，新增设施农业1万亩，新建粮食生产功能区10万亩以上。规划建设重要农产品保护区、农业产业集聚区和现代特色农业强镇。鼓励工商资本投资农业，引进农业大项目，促进土地集中连片规范有序流转。大力推进农业产业融合，加快花卉苗木等农业主导产业发展，提升畜禽养殖业水平。认真抓好“米袋子”、“菜篮子”工程。建成市农产品质量安全监测中心，新增“三品一标”认证和登记保护50个。加快金华农产品批发市场项目建设，办好中国苗交会、义乌森博会、武义养博会、磐安药交会等农业展会。

深化建设美丽乡村。新建美丽乡村风景线10条、精品村20个、秀美村50个。积极争创国家级、省级湿地公园，支持浦江建设国家湿地公园。重视历史文化村落保护利用，强化古村落古建筑、古道古桥、古树名木保护，编好“百村故事”，开展徐霞客游线标志地保护和申遗工作。

努力增加农民收入。完善农民增收政策体系，实施新型职业农民培育计划，加大农民就业技能培训力度，鼓励返乡农民、有志青年开展农业创业，完成“农村实用人才培育计划”1万人以上。深化农村“三权”改革，加快户籍制度改革。大力发展农村电商，促进农民增收。

（六）加强生态环境建设。推进环境整治从重点突破向综合治理转变，切实提高环境质量。

强化水环境综合治理。全面落实河长制，加快城镇截污纳管扩面提速和排污口整治，实施城镇“六小”服务业综合整治，推行污水处理“金华标准”，推进城镇污水处理厂“一厂一湿地”生态治水建设。消灭25条市级主要支流入江断面劣V类水质，落实“清三河”长效保洁措施，防范回潮反弹，兑现“三年可游泳”承诺。

强化大气环境综合治理。深化治气三年行动，加快推进燃煤电厂以及水泥、热电等重点行业超低排放或清洁排放技术改造。推进重点行业挥发性有机污染物治理，强化机动车排气污染防治，加强施工和道路扬尘防控，严格餐饮油烟、装修和干洗废气控制，禁止秸秆焚烧，有序推进烟花爆竹禁燃禁售工作，力争PM2.5平均浓度下降20%。

强化城乡环境综合治理。完成拆违500万平方米、“三改”540万平方米任务。深化“两路两侧”、“四边三化”整治，基本消除主要道路沿线赤膊房，改善农村道路沿线、房前屋后、池塘沟渠综合环境。农村和城镇生活垃圾分类减量处理覆盖率分别达到95%和60%以上。健全治堵治超长效机制，改善群众出行条件。

（七）改善民生福祉。坚持发展为了群众，守住民生底线，重视解决人民群众最关心最直接最现实的利益问题。

促进居民就业增收。实施“互联网+就业”服务新方式，大力发展众创、众包、众扶、众筹空间，做好高校毕业生等各类群体就业。完善社会平均工资指导线确定机制，推行企业工资集体协商制度，确保劳动报酬和劳动生产率同步提高。加大精准扶贫力度，做好低收入群体增收帮扶工作，实现低收入农户人均收入增长15%以上。大力支持武义、磐安“摘帽快跑”、绿色崛起。

完善社会保障体系。实施全民参保计划，健全“五费合征”、“网上申报”等工作机制，加快推进机关事业单位养老保险制度改革，养老、医疗等五大保险参保规模达630万人次。重视社会养老工作，促进养老机构规范发展，提升养老服务水平。

提高公共服务的普惠性。争创基本实现教育现代化县2个以上，改善薄弱学校基本办学条件13所，建设信息化特色学校50所，创建省普通高中特色示范校10所。重视发展学前教育、职业教育、高等教育和特殊教育。打好医改联动“组合拳”，深化“双下沉、两提升”，完善社区卫生服务体系，加强智慧医疗建设。加快市人民医院PPP项目建设，启动实施市妇幼保健院等迁建工程。实施“全面两孩”政策，强化妇幼健康服务，提升出生人口素质。加快公共文化服务体系标准化建设，重视非物质文化遗产传承保护利用和农村文化礼堂建设，完成第一次全国可移动文物普查工作。加快体育设施向公众开放，构建全民健身服务体系。

创新社会治理。围绕G20峰会等重大活动，全面加强信访、反恐、维稳、应急管理等工作，从严从重打击黑恶势力、暴力犯罪等各类违法犯罪行为。深入推进基层平安创建活动，完善立体化治安防控体系，婺城、武义、磐安争创“平安金鼎”。加强重点行业、重点领域安全隐患排查整治，探索“互联网+安全”监管方式，提升安全生产治理能力。全面开展地质灾害隐患排查和整治。深化8890便民服务，推出创业就业、健康服务等特色项目。加强虚拟社会管理，提高网络舆情引导能力。完善流动人口和特殊人群管理服务。

同时，加强工会、共青团、妇联等人民团体工作，重视做好民族宗教、对台、民防、气象、统计、档案、国防、双拥、地方志、残疾人和关心下一代等工作。

　四、建设人民满意的服务型政府

　　全面实施“十三五”规划，确保完成今年各项目标任务，对政府自身建设提出了新的更高要求。

　（一）坚持依法行政。制定实施法治政府建设实施纲要，坚持市政府常务会议学法、政府部门夜读学法制度，认真执行行政负责人出庭应诉制度，完善人大代表政协委员列席市政府常务会议制度。自觉接受人大依法监督、政协民主监督，主动接受司法监督、社会监督和舆论监督，加强政府内部层级监督和专门监督，推进审计监督全覆盖。全力支持配合市人大及其常委会开展地方立法工作，做好规范性文件和政策性文件清理工作。完善政府信息公开机制，全面实施行政执法责任制和行政综合执法，加快行政复议体制改革，积极推进阳光执法、网上执法。

（二）强化责任担当。大力弘扬金义综保区申报建设工作体现的“金华精神”、浙师大截污纳管大会战展现的“金华力量”、华科汽车建设创造的“金华速度”，始终保持“马上就办、只争朝夕”的责任感和紧迫感，始终保持“五加二、白加黑”的工作热情，始终保持“勇于创新、一线攻坚”的科学方法，做到困难面前不退缩、问题面前不回避，接受任务不找借口、执行任务不讲困难，以舍我其谁的担当、虎口夺食的勇气、“店小二”的服务，甩开膀子，大干五年。

（三）加强廉政建设。认真贯彻《准则》和《条例》，严格遵守政治纪律、组织纪律、廉洁纪律、群众纪律、工作纪律、生活纪律，健全比学赶超、求严求实的工作机制，着力破解“为政不为”问题。严格落实反腐倡廉“一岗双责”制度，建立廉政建设与各项工作融合机制，强化源头防腐。完善厉行节约长效机制，实现“三公”经费支出零增长。基本完成公务用车制度改革。全力做好省委巡视组巡视发现问题的整改落实工作，坚决防止“四风”反弹。

各位代表，今年我们按照民生实事“群众提、大家定”的理念，确定了十个方面实事。市政府将进一步健全工作机制，切实把民生实事办好。

1. 加大雾霾治理力度。完成浙能兰溪电厂2台机组烟气超低排放技术改造，淘汰高污染燃料锅炉2000台以上，县级以上城市建成区全面禁止使用高污染燃料锅炉。新建新能源汽车充换电站7座以上，充电桩500个以上。全面淘汰黄标车。

2. 加大治污清淤力度。完成4座镇级污水处理厂一级A提标改造，新增污水管网280公里，实施农村生活污水治理800个行政村，新增受益农户10万户，完成河道清淤等综合整治200公里以上。

3. 提升教育均衡水平。启动创建义务教育标准化学校35所，其中建成9所。建成中小学塑胶跑道18条，覆盖率达到90%。等级幼儿园、普惠园覆盖面分别达到92%和80%以上。

4. 方便群众就医。深化医保付费方式改革，在金华市区7家主要医疗机构探索“同病同价、优质优价”结算方式。基层责任医生规范签约率达20%。实现在职职工住院医疗互助保障参保人数30万人。

5. 提高就业养老服务水平。新增城镇就业7万人，城镇登记失业率控制在4%以内。新建城乡社区居家养老服务照料中心100个，建设医养结合和家院互融养老服务机构20家，新增养老床位（含社会床位）2000张以上。

6. 加强食品安全监管。创建放心农贸市场20家，城区农贸市场质量追溯体系建成率达75%以上，建成“阳光厨房”200家。新增二维码可追溯农业企业、合作社、家庭农场350家。

7. 加强农村惠民服务。新增农村文化礼堂（文化公园）100个。提升美丽乡村公路1000公里，改善10万农村人口饮水条件。完成农村困难家庭危房改造1000户以上。新建示范农村电商服务站320个，建成示范电商专业村100个。

8. 提升城乡卫生环境。农村生活垃圾分类减量处理实现乡镇全覆盖、行政村覆盖95%以上。实施农村环境“四整治一美化”行动，创建达标村60%以上。新建或改造旅游厕所229个。

9. 深入推进城市治堵工作。开通运行BRT3号、4号线，新增公共自行车桩位3000个。打通断头路22条，道路优化改造15条，金华市区公交分担率比上年提高2个百分点。

10. 推进智慧城市建设。金华市区发放社会保障•市民卡20万张。全市建成无线局域网（WiFi）AP点30000个，并免费开放。新建城市社区智能投递终端260个。

各位代表，干在实处永无止境，走在前列要谋新篇。让我们切实肩负起时代和人民赋予的神圣使命，在中共金华市委的坚强领导下，振奋精神、团结拼搏、勇于担当、真抓实干，为“走在前列、共建金华”而努力奋斗！
